# Spatial concepts of number, size, and time in an indigenous culture

**DOI:** 10.1126/sciadv.abg4141

**Published:** 2021-08-11

**Authors:** Benjamin Pitt, Stephen Ferrigno, Jessica F. Cantlon, Daniel Casasanto, Edward Gibson, Steven T. Piantadosi

**Affiliations:** 1 Department of Psychology, UC Berkeley, Berkeley, CA, USA.; 2 Department of Psychology, Harvard University, Cambridge, MA, USA.; 3 Department of Psychology, Carnegie Mellon University, Pittsburgh, PA, USA.; 4 Department of Human Development, Cornell University, Ithaca, NY, USA.; 5 Department of Brain and Cognitive Sciences, MIT, Cambridge, MA, USA.

## Abstract

In industrialized groups, adults implicitly map numbers, time, and size onto space according to cultural practices like reading and counting (e.g., from left to right). Here, we tested the mental mappings of the Tsimane’, an indigenous population with few such cultural practices. Tsimane’ adults spatially arranged number, size, and time stimuli according to their relative magnitudes but showed no directional bias for any domain on any spatial axis; different mappings went in different directions, even in the same participant. These findings challenge claims that people have an innate left-to-right mapping of numbers and that these mappings arise from a domain-general magnitude system. Rather, the direction-specific mappings found in industrialized cultures may originate from direction-agnostic mappings that reflect the correlational structure of the natural world.

## Teaser

Among the Tsimane’ people of Bolivia, concepts of number, size, and time have spatial structure but no consistent direction.

## INTRODUCTION

In many cultures, people reason about number, time, and other conceptual domains using space. For example, Americans and Europeans implicitly associate smaller numbers with the left side of space and larger numbers with the right side, forming an implicit “mental number line” (*1*, *2*). Although the mental number line has been the subject of hundreds of studies (*3*), its phylogenic origins remain controversial. Some scholars have proposed that a left-to-right mapping of number may be innate, based on studies of human infants and nonhuman animals like monkeys, chicks, and fish (*4*–*8*). For example, after habituating to visual arrays with a fixed number of dots, human neonates looked to the left in response to smaller numbers of dots and to the right in response to larger numbers (*9*). Innate left-to-right mappings may not be unique to number (*10*); on some accounts, number shares representational structure with other conceptual domains, including time and size, as part of a “generalized magnitude system” in parietal cortex (*11*–*14*). On the strongest versions of this theory, the mental number line is a manifestation of a domain-general “mental magnitude line” that provides common spatial structure to mappings of number, size, and time, among others (*15*, *16*). If so, then any spatial biases in number mappings should also be found for mappings of these other domains.

These proposals are difficult to test in industrialized groups, where any innate spatial biases may be masked by cultural conventions that cause number, time, and other domains to be spatialized in a consistent direction (e.g., reading and counting from left to right). These conventions are enough to influence mental mappings of these domains, which vary in direction with spatial practices like reading (*17*–*19*) and which can be independently changed by brief spatial training (*20*, *21*). These effects support theories of metaphorical mental representation positing that each mental mapping arises from the correlations between the relevant domains (e.g., space and number; space and time) that are found in the natural and cultural environment (*21*–*23*). On this alternative account, direction-specific cultural experiences act on mental mappings that initially lack specific direction (*20*).

To isolate effects of cultural experience, we tested mental mappings in the Tsimane’, an indigenous group of farmer-foragers who live in the Amazon basin of Bolivia (*24*). With little or no formal education, low levels of literacy and numeracy, and few modern technologies (*25*), many Tsimane’ adults have scant experience with the directional practices that may mask innate spatial biases in industrialized cultures. The Tsimane’ therefore allowed us to study the origins of mental mappings, in two ways. First, if mental mappings of number (or other domains) have rightward direction by default, then unindustrialized groups like the Tsimane’ should show this spatial bias (*26*–*29*). Second, if mental mappings inherit “a common pattern of spatial organization” (*15*) from a mental magnitude line, then a given person should map different domains (e.g., number, time, size) in the same direction (e.g., all right-to-left), even if that direction differs across individuals (*11*, *16*).

## RESULTS

### Experiment 1: Number and size mappings on the lateral axis

**Fig. 1:**
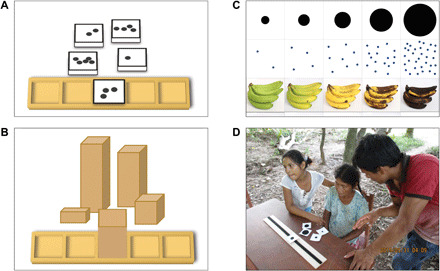
Materials for mapping tasks. In experiment 1 (left), participants arranged stimuli that varied in (**A**) number and (**B**) size. In experiment 2 (right), participants arranged (**C**) size, number, and time stimuli on all three spatial axes. (**D**) A translator explains the size task (on the lateral axis) to a Tsimane’ woman.

In experiment 1, we tested number and size mappings in Tsimane’ adults (*N* = 96; ages 17 to 78) by having them manually arrange stimuli that varied in number (1 to 5 dots) or size (1 to 5 inches) along a lateral response array, as shown in Fig. 1 (left). We included comparison groups of U.S. adults (*N* = 18; ages 29 to 50), who have extensive experience with left-to-right cultural practices, and of U.S. children (*N* = 31; ages 3:1 to 5:5), who have relatively little such experience. If people have an innate predisposition to map numbers or other magnitudes from left to right, then those spatial biases should be evident in all three groups.

**Fig. 2:**
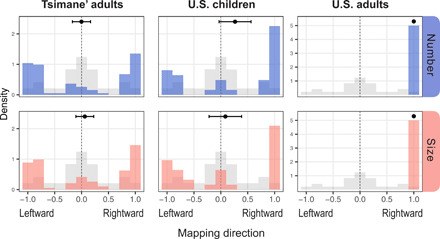
Lateral number and size mappings in three populations. Mappings ranged from perfectly leftward to perfectly rightward, with less orderly mappings in the middle. Colored bars show participants’ mappings of number (blue) and size (pink), which differed significantly from chance (gray). Black dots and whiskers show mean mapping scores and uncorrected 95% confidence intervals.

Figure 2 shows the distribution of mapping scores that each group (columns) produced for each domain (rows), contrasted with the chance distribution (in gray). U.S. adults showed a strong rightward bias in their mappings; both number and size increased monotonically from left to right in every case. By contrast, Tsimane’ adults and U.S. children showed no such bias for number or size (whether these domains were analyzed separately or pooled together). Rather, participants were equally likely to map the stimuli to the left as to the right, as shown by their response histograms with peaks near the extremes. The dot and whiskers on each plot show that, on average, these mapping scores did not differ significantly from zero. We found the same result when analyzing only the mappings with clear directionality, excluding mappings with scores near zero. Only the most educated of our Tsimane’ participants showed any sign of a rightward bias in their mappings (see the Supplementary Materials).

In principle, participants could have shown no systematic use of space at all, arranging the stimuli without respect to their relative magnitudes. This was not the case; for every group and domain, the observed distribution of mappings (i.e., colored bars) differed significantly from the chance distribution (i.e., gray bars). For example, whereas randomly arranging the stimuli would result in a perfectly monotonic array only 1.5% of the time by chance, these perfectly ordered arrays occurred 47 to 100% of the time in our participant groups. Therefore, the lack of directional bias in U.S. children and Tsimane’ adults cannot be explained by a misunderstanding of the task or by an absence of spatial associations; both groups systematically ordered the stimuli according to their relative magnitudes, in one direction or the other.

These findings challenge claims that people have an innate predisposition for left-to-right mappings of magnitudes generally (*15*) or of numbers specifically (*9*). If children started with an innate rightward bias, then enculturating them with clear rightward spatial conventions should only strengthen these mappings (*30*). Yet, here, U.S. children showed no rightward bias, despite the spatial conventions of American culture (*30*–*32*). Likewise, if direction-specific cultural conventions changed or masked innate spatial biases (*5*), then people without such conventions should retain those biases. Yet, here, Tsimane’ adults showed no evidence of a default rightward bias in their mappings (*26*). Therefore, the lack of directional biases among U.S. children and Tsimane’ adults cannot reflect displacement of innate rightward biases. Rather, these findings support the proposal that the direction of lateral mappings is determined by direction-specific experiences, like those that are common in industrialized cultures (*21*, *22*).

### Experiment 2: Number, size, and time mappings on three axes

Even if the hypothesized mental magnitude line does not have any default direction, it could nonetheless provide a common spatial structure to different mappings in a given mind. This is one of the “strong predictions” (*11*) of the generalized magnitude system proposal; different mappings should share a common direction (*15*, *16*). To clarify the links between different mappings in a given mind, experiment 2 tested a new group of Tsimane’ adults (*N* = 60; ages 12 to 86) on their mappings of all three of the magnitude domains central to the generalized magnitude system proposal—size, number, and time (*16*)—as shown in Fig. 1C. We also expanded our testing to include all three spatial axes—lateral, vertical, and sagittal (i.e., front-back)—where mental mappings of number (and other domains) have been observed (*33*–*35*). In principle, the lack of directional bias observed in experiment 1 could be unique to left-right space, which is more difficult for people to distinguish than up-down or front-back space (*36*–*38*) and which may be especially difficult in unindustrialized groups like the Tsimane’ (*39*, *40*). We therefore tested participants’ mappings of all three conceptual domains on all three spatial axes. This fully within-subject design allowed us not only to compare mapping direction across individuals (*26*, *27*, *35*) but also across domains within individuals. To ensure that participants could accurately compare the magnitudes of each stimulus set as intended, we asked them to identify which stimulus in each set was biggest/most/oldest and which was smallest/least/newest and we excluded mappings (12%) for which participants did so incorrectly.

**Fig. 3:**
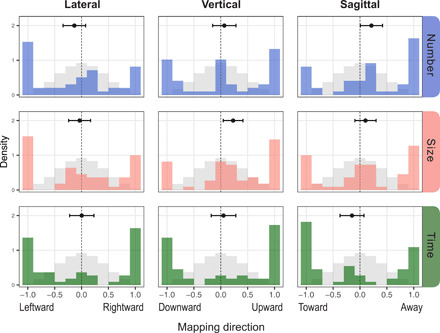
Number, size, and time mappings on three spatial axes. Mappings ranged from perfectly negative (i.e., leftward, downward, or toward) to perfectly positive (i.e., rightward, upward, or away), with less orderly mappings in the middle. Colored bars show participants’ mappings of number (blue), size (pink), and time (green), which differed significantly from chance (gray). Black dots and whiskers show mean mapping scores and uncorrected 95% confidence intervals.

Figure 3 shows the distribution of mappings that participants produced for each domain on each spatial axis. As in experiment 1, participants’ mappings (i.e., colored bars) differed significantly from the chance distribution (i.e., gray bars), indicating that participants arranged the stimuli in space according to their relative magnitudes, as instructed. Yet, despite this systematicity, they showed no directional biases on any spatial axis for any domain (whether number, size, and time were analyzed separately or pooled together), replicating the results of experiment 1 and extending them to the domain of time and to the vertical and sagittal axes. The dots and whiskers in Fig. 3 show the means and uncorrected 95% confidence intervals (CIs) of each mapping on each axis; none reliably differed from zero (when correcting for multiple comparisons; see the Supplementary Materials). A subset of participants produced mappings with scores near zero, perhaps because they misunderstood our task instructions; given that any demonstration of the tasks would have biased responses, our explanations were purely verbal and communicated via Spanish-Tsimane’ interpreters. In principle, these intermediate scores could mask a directional bias in more systematic mappings. We therefore reanalyzed the data using only mappings with clear directionality (i.e., excluding mappings with scores near zero). We found the same pattern of results: For all three domains on all three axes, the Tsimane’ used space systematically but showed no directional biases.

**Fig. 4:**
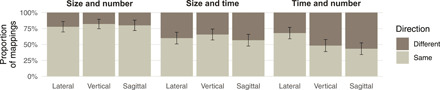
Within-participant consistency of mapping direction for each pair of domains. Error bars show the SEM.

Using this same subset of mappings, we evaluated directional consistency within participants by doing pairwise comparisons among the three mappings each participant produced on a given axis, as shown in Fig. 4. If these mappings were manifestations of a single mental magnitude line, then any coherent mappings that a participant produced should have gone in the same direction. Contrary to this prediction, participants mapped all three domains in the same direction in only 42% of cases (95 % CI : 0.32 to 0.54); in all other cases, participants arranged stimuli systematically (i.e., in order) but in opposite directions on a given axis: Rightward versus leftward, upward versus downward, or toward versus away. Across axes, number and size mappings were most consistent in direction (80% same direction; 95 % CI : 0.70 to 0.87), whereas size and time patterned together in 61% of cases (95 % CI : 0.51 to 0.70) and time and number patterned together in only 53% of cases (95 % CI : 0.42 to 0.63). It may be unsurprising that number and size mappings were the most aligned, given that these domains are correlated in the natural world [i.e., greater numbers of objects occupy more space (*41*)] as in our experimental stimuli (but see Materials and Methods) and that the Tsimane’ describe numbers in terms of size in their language, as in English (i.e., a “big” or “small” number; see the Supplementary Materials). Nevertheless, participants produced mappings with opposing directions for every set of domains on every axis (Fig. 4).

## DISCUSSION

In two experiments, we tested the mental mappings of three conceptual domains (i.e., size, time, and number) on three spatial axes (i.e., lateral, vertical, and sagittal) in three populations (i.e., U.S. children, U.S. adults, and Tsimane’ adults). The results challenge existing theories of mental mappings, in two ways. First, our results are at odds with the proposal that people are born with a left-to-right mapping of number, as suggested by behavioral effects in human infants and nonhuman animals (*4*–*8*). When explicitly instructed to spatialize stimuli on the basis of number, only U.S. adults showed a left-to-right directional bias; U.S. children and Tsimane’ adults made mappings that, although highly systematic, were no more likely to increase to the right as to the left. This pattern, which we found in three independent samples, is consistent with previous findings in unindustrialized adults (*26*, *29*) but contrasts with studies of infants and nonhuman animals, who appear to show a rightward bias. These different effects are likely due to substantial differences in methodology (i.e., studies of infants and animals cannot instruct their participants to respond on the basis of number) and therefore may reflect different cognitive processes. For example, some scholars have suggested that the effects in infants and nonhuman animals may reflect hemispheric specialization for spatial frequency (*42*) or emotional motivation (*8*) rather than spatial-numerical associations.

Second, the mappings we observed cannot be explained by a mental magnitude line, even one without a default direction. If different mappings reflected “the same coordinate system applied to all magnitudes” (*12*), then any coherent spatial mappings should have had the same direction in a given mind (*11*, *15*, *16*). They did not; even the most systematic mappings of size, time, and number regularly went in different directions in the same participant. Therefore, although a generalized magnitude system may explain why people tend to associate more in one domain with more in another (*43*, *44*), it does not explain the way people map these domains onto space (*45*).

Why did participants’ mappings have coherent spatial structure but lack consistent direction? In principle, each person could have an idiosyncratic set of mappings that are stable over time (e.g., always rightward for size and leftward for number), but it is unclear why their directions would vary across domains and individuals, given the shared language and culture of our participants. Rather, each mapping may begin without specific direction, leading people to map a given domain in different directions at different times. According to a theory of metaphorical mental representation (*22*), such direction-agnostic mental mappings arise from cross-domain correlations that are observable in the natural world, like that between space and time: As objects travel further in space, more time passes. Because this correlation applies equally in all directions, it yields mental mappings in all directions. From this direction-agnostic starting point, cultural experiences like reading text or solving equations then provide space-time and space-number correlations that are direction-specific (e.g., left to right), yielding the direction-specific mental mappings observed in industrialized groups (*20*, *21*, *46*). In this way, culture-specific mental mappings like the left-to-right mental number line may originate from multidirectional mappings that reflect the correlational structure of the natural world.

## MATERIALS AND METHODS

### Experiment 1

#### Participants

Tsimane’ adult participants were 96 adults (age range: 17 to 78 years; mean age = 31.63 years; 61 females; schooling range: 2 to 11 years; mean schooling = 7.59 years). Participants were recruited from Tsimane’ communities near San Borja, Bolivia. All guidelines and requirements of the University of Rochester’s Research Subjects Review Board were followed for participant recruitment and experimental procedures. Interpreters were provided by the Centro Boliviano de Investigación y de Desarrollo Socio Integral.

U.S. children participants were 31 children (age range: 3:1 to 5:5; mean age = 4:3; 22 females). Participants were recruited through the Kid Neuro Lab at the University of Rochester. All guidelines and requirements of the University of Rochester’s Research Subjects Review Board were followed for participant recruitment and experimental procedures.

U.S. adult participants were 18 adults (age range: 29 to 50 years; mean age = 36.17 years; 17 females), who were the parents of the child participants. All guidelines and requirements of the University of Rochester’s Research Subjects Review Board were followed for participant recruitment and experimental procedures.

#### Materials

We constructed two sets of five wooden blocks—one set for the number task and one set for the size task (see Fig. 1, A and B). In the number task, the blocks were all the same height and were painted white. Each of the number blocks had a number of uniform sized black dots (from 1 to 5 dots) positioned randomly on the top face. In the size task, the blocks varied in height (from 1 to 5 inches in increments of 1 inch). All of the blocks in both conditions were 2 inches wide by 2 inches deep. A wooden board with five equally spaced depressions (^1^/_4_ inch deep) was constructed such that each of the five blocks could be placed securely in a depression.

#### Procedure

Each participant performed two tasks (size and number) on the lateral axis. The order of the tasks was counterbalanced across participants. For each task, participants were seated directly across from the experimenter. The wooden board was placed in front of the participant, and the five blocks in the first task were presented just behind the board in a random array.

For each task, the experimenter picked up the medium block (3 dots or 3 inches tall) and placed it in the middle position on the board. As the experimenter placed the block, the subject was told “This is the 3rd block. It goes here.” The experimenter then picked up the first block (1 dot or 1 inch tall) and handed it to the subject directly over the center of the board and said, “This is the first block. It goes in the first position.” Participants then placed the block on the board without feedback. This procedure was repeated for the second, fourth, and fifth blocks, until all positions were filled. The order of the tasks was counterbalanced across participants.

### Experiment 2

#### Participants

Sixty Tsimane’ adults (age range: 12 to 86 years; mean age = 39.18 years; 30 females; schooling range: 0 to 13 years; mean schooling = 3.12 years) provided informed consent and participated in exchange for goods. All protocols were approved by the Institutional Review Board of UC Berkeley.

#### Materials

We constructed three sets of five cards, one set for each of the three tasks (see Fig. 1C). In the size task, each card showed a single black circle whose area differed by factors of two. In the number task, each card showed an array of dots (i.e., a “dot cloud”), and the number of dots differed across cards by factors of two, from 2 to 32. In the time task, cards depicted a bunch of bananas of various ages, from underripe to rotten. A subset of participants (*N* = 18) received an alternative set of time in which time was conveyed by an aging face and an alternative set of number stimuli in which dot clouds had constant total area (i.e., the more dots, the smaller the dots; see fig. S1). Mapping direction did not differ as a function of stimulus variant for either number or time (*P*s > 0.5), so these variants were pooled and analyzed together. On the back of each card (approximately 3 × 3 inches, laminated), we placed an adhesive piece of Velcro that allowed the card to be temporarily affixed to a Velcro board. Velcro boards were 3 × 24 inches and had a strip of Velcro adhered lengthwise along the middle of the board. Three identical Velcro boards were used, one for each spatial axis (to avoid translating a single board between spatial axes in view of participants).

#### Procedure

Before completing the spatialization tasks, a subset of participants performed simple tests of their literacy and numeracy. In the literacy test, participants were asked to read aloud a series of 10 common Spanish words, which were printed in large typeface. Their reading score is the number of these words they correctly read aloud. In the numeracy test, they were asked to solve a series of 12 simple arithmetic problems, which were presented to them on paper (and read aloud). Their math score is the number of these problems they answered correctly, without help.

Each participant performed all three tasks (size, time, and number) on each spatial axis (lateral, vertical, and sagittal) before progressing to the next axis, for a total of nine trials. The order of spatial axes was counterbalanced across participants, and the order of tasks on a given axis was quasi-randomized.

Participants were seated at a table between the experimenter and translator (such that all three were on the same side of the table). Two of the Velcro boards were positioned on the table, one oriented laterally to the participant and the other oriented sagittally. The third Velcro board was oriented vertically (i.e., resting on its short side) on a chair beside the participant.

For each task, participants were presented with all five cards, which the experimenter placed in a disordered pile on the table or chair near the relevant Velcro board. After explaining how the five cards of the given set differed from each other (in size, age, or number), the experimenter picked up the card depicting the “medium” magnitude and placed it in the middle of the Velcro board (and explained this action verbally, without naming the card’s ordinal position; see Fig. 1D). Participants were instructed to “organize the other cards on the board in order of their size/age/number.” Once the participant was done placing all the cards on the board, the experimenter noted the position of each card, without providing any feedback to the participant. After each task on the first axis, the experimenter also asked the participant to indicate which card had the smallest circle, the newest bananas, or the fewest dots and then to indicate which card had the largest circle, the oldest bananas, or the most dots. All cards were removed from the Velcro board after each trial. After completing all three tasks on a given axis, the Velcro board for that axis was removed from sight, and the participant was directed to the board that corresponded to the next axis.

## SUPPLEMENTARY MATERIALS

Supplementary material for this article is available at http://advances.sciencemag.org/cgi/content/full/7/33/eabg4141/DC1

## Appendix

View/request a protocol for this paper from *Bio-protocol*.
